# Partition of the Reactive Species of the Suzuki–Miyaura Reaction between Aqueous and Micellar Environments

**DOI:** 10.1021/acs.jpcb.2c04591

**Published:** 2022-11-04

**Authors:** Anna Ranaudo, Claudio Greco, Giorgio Moro, Anita Zucchi, Sara Mattiello, Luca Beverina, Ugo Cosentino

**Affiliations:** † Department of Earth and Environmental Sciences, University of Milano-Bicocca, Piazza della Scienza 1, Milan 20126, Italy; ‡ Department of Biotechnology and Biosciences, University of Milano-Bicocca, Piazza della Scienza 2, Milan 20126, Italy; § Department of Materials Science, University of Milano-Bicocca, Via Roberto Cozzi 55, Milan 20125, Italy

## Abstract

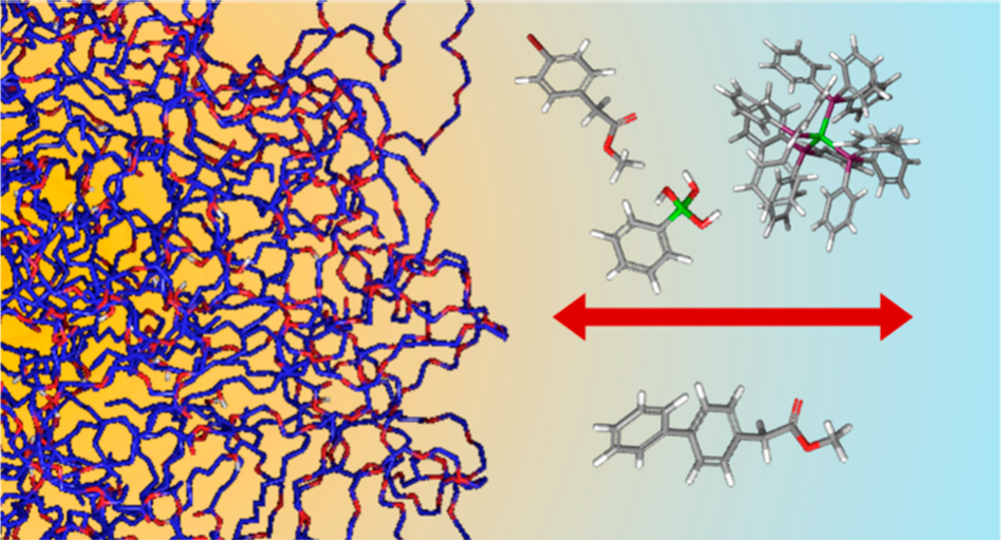

The Suzuki–Miyaura reaction between the aryl halide (**1**) and the phenyl boronic acid (**2**), in the presence of the palladium(0) complex (**3**) as catalyst, gives the cross-coupling product (**4**) in quantitative yield when performed in basic aqueous solution of the nonionic surfactant Kolliphor-EL (K-EL). The partition between the aqueous and micellar environments of the species of this reaction has been investigated by means of Molecular Dynamics (MD) simulations. Starting from the K-EL molecules dispersed in water, a micelle model has been generated by MD simulations, adopting the 2016H66 force field. Reagent and product species have been described with the same force field, once the reliability of this force field has been tested comparing the *n*-octanol/water partition free energies calculated from the MD and Free Energy Perturbation (FEP) method with those obtained from the quantum-mechanical SMD method. The potential of mean force for the transfer process between water and the micellar phase of the different species has been calculated by the MD simulations and the Umbrella Sampling (US) method. The overall picture that emerges from these results confirms that the molecular species involved in this reaction prefers the micellar environment and concentrates in different but close zones of the micelle. This supports the experimental evidence that the use of suitable surfactant agents promotes reactivity, allowing micelles to behave as nanoreactors in which reactive species are solubilized and enhance their local concentration.

## 1 Introduction

In the framework of a sustainable approach to chemical processes and products, the green chemistry principles^1^ are highlighted as a relevant strategy to adopt a proper solvent selection to minimize toxicity, energy demand, and pollution. Water is the green solvent for excellence, but being a poor solvent for most organics, it is of limited use in synthesis. However, the use of suitable surfactant agents under micellar conditions allows micelles to work as nanoreactors capable of solubilizing and enhancing the local concentration of the reactive species, favoring reactivity.

The palladium-catalyzed Suzuki–Miyaura reaction is one of the most efficient reactions for the synthesis of unsymmetrical biaryls ArAr′ from aryl halides ArX and arylboronic acids Ar′B(OH)_2_ using a palladium catalyst and a base. The mechanism has been elucidated, and it involves oxidative addition of palladium catalyst to the halide and subsequent transmetalation and reductive elimination steps that provide the product.^2^ This reaction that is generally performed in organic solvents can be also carried out in water in the presence of suitable surfactants, at room temperature under oxygenated environment, with significantly shortened reaction times in comparison to standard methods.^3,4^

Despite the wide applications of micellar conditions in organic synthesis, a deeper insight at the molecular level of the partition distribution between the aqueous and the micellar phase of the species involved is still lacking. Theoretical methods represent an essential tool to reach this target. Among these, Molecular Dynamics simulations combined with the Umbrella Sampling (US)^5,6^ or the COSMOmic^7^ method, an extension of the COSMO-RS approach,^8^ are able to take into account the anisotropic environment of the micelle. Both methods calculate the free energy profile as a function of the distance of a solute from the micellar environment, thus providing detailed information about the partition behavior of the solute.

MD simulations have been extensively applied to the study of partition in lipid bilayers, providing information, for instance, in drug–membrane partition studies.^9−11^ The combination of the MD and COSMOmic methods has been successfully applied to predict partition equilibria of various solutes in biomembranes^12−14^ and in different micellar systems.^15−20^ However, to the best of our knowledge, no studies have been yet performed on the partition between the aqueous and micellar environments of species involved in the reactive processes.

**Scheme 1:**
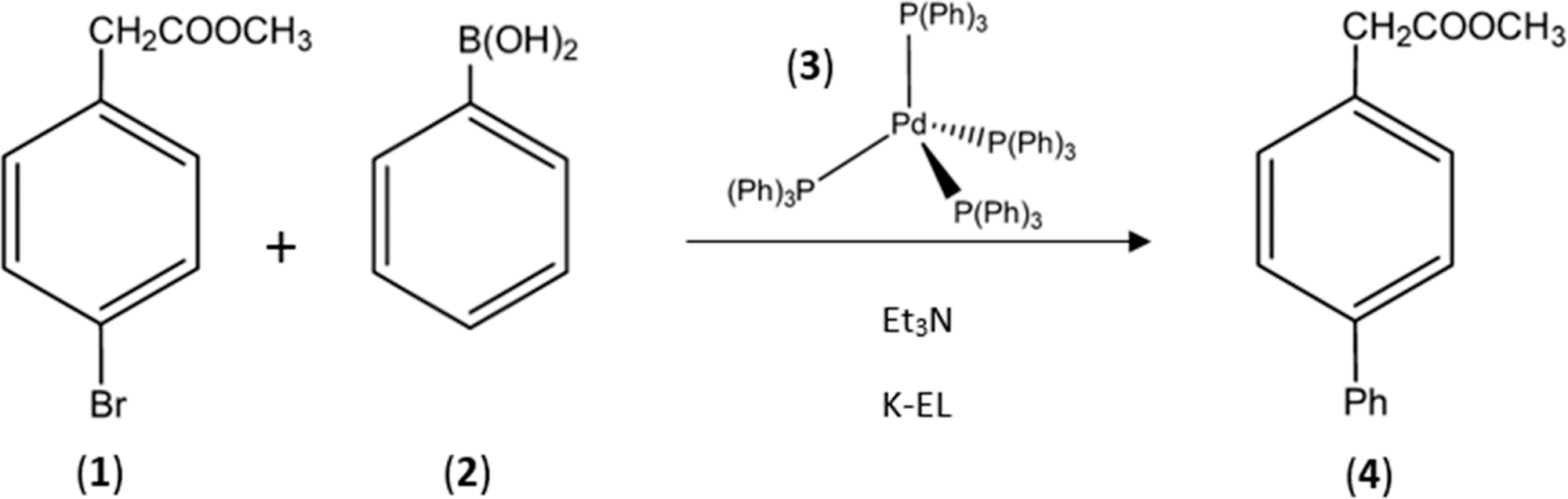
Considered Suzuki–Miyaura Cross-Coupling Reaction

Here, we present the Suzuki–Miyaura cross-coupling synthesis of the biaryl product (**4**) obtained from the aryl halide (**1**) and the phenyl boronic acid (**2**), with the palladium(0) complex (**3**) as catalyst: the reaction has been carried out in aqueous solution and in the presence of triethylamine (Scheme 1), which activates the boronic acid in the form of the corresponding hydroxyborate. In the basic aqueous phase, the phenylboronic acid is highly soluble (the predicted value from the SciFinder database^21^ at 25 °C and pH 10 is 2.45 mol L^–1^), while methyl 4-bromophenylacetate presents low water solubility (the predicted value from the SciFinder database^22^ at 25 °C and in the pH 1–10 range is 3.1 × 10^–3^ mol L^–1^); thus, the presence of a surfactant is required to allow the reaction to proceed. In our case, the reaction has been carried out in the presence of the nonionic surfactant Kolliphor-EL (K-EL).

The aim of our investigation concerned the study by a computational approach of the partition free energies between water and the micellar environment of the species involved in this reaction.

**Figure 1:**
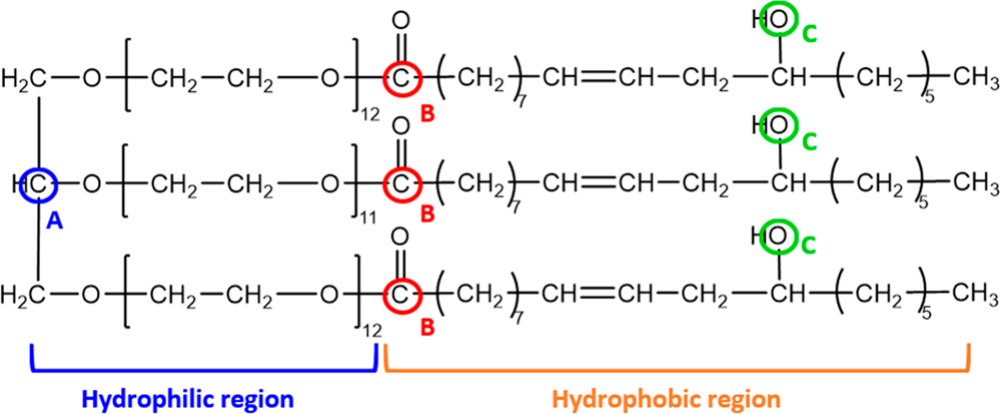
Chemical structure of the most abundant constituent present in the K-EL mixture. A, B, and C highlight the atoms selected to label the hydrophilic, interface hydrophilic–hydrophobic, and hydrophobic regions.

K-EL belongs to the class of nonionic surfactants, and it is a triricinoleate ester of ethoxylated glycerol. It derives from the reaction of ethylene oxide with castor oil, and it is constituted by a complex mixture. The most abundant constituent of such a mixture presents the chemical structure reported in Figure 1, which is the K-EL component considered in the study reported in ref (23) and the one chosen for this work.

The chemical-physical properties of K-EL colloids have been investigated either experimentally or by MD simulations:^23^ the computational models, based on the 2016H66 force field,^24,25^ were able to reproduce the main characteristics of the K-EL micelle. The 2016H66 force field encompasses the Gromos 53A6;^26^ the Gromos 53A6OXY,^27^ a reoptimization of 53A6 based on compounds containing oxygen; and the 53A6OXY+D force field,^28^ which has been parametrized with the inclusion of vicinal diether functions, which are contained in the K-EL molecule.

According to the experimental evidence, the micelle models obtained by the MD simulations^23^ present a spherical morphology, with aggregate size in the 12–15 nm experimental range; moreover, the assembly of the K-EL molecules within the micelles shows the fatty acid chains located in the micellar core, reproducing the core–shell structure experimentally observed.

In our investigation, the 2016H66 force field has been adopted to model both the reactive species and the micelle. Before studying the micelle/water partition, we preliminarily investigated the reliability of this force field in reproducing the *n*-octanol/water (o/w) partition free energy, calculated from the solvation free energy of the reactive species in the two solvents. *n*-Octanol is considered a representative model of the amphiphilic environment of lipid bilayers and, more generally, of an amphiphilic micellar environment. Solvation free energies have been calculated by using MD simulations and the Free Energy Perturbation (FEP) method.^29−31^ As experimental data on the solvation free energies and o/w partition free energies of the considered molecules are lacking, the MD/FEP values have been compared to the corresponding quantities calculated at the quantum mechanical level by the Solvation Model Density (SMD) method,^32^ which we considered as reference values. The potential of mean force for the transfer process between water and the micellar phase of the different species has been calculated by MD simulations and the Umbrella Sampling (US) method.

The organization of this Article is as follows: (i) comparison of the *n*-octanol/water partition free energy values calculated by MD/FEP and SMD; (ii) description of the K-EL micelle computational model setup and characterization of the micellar environment; and (iii) potential of mean force for the simulations of the transfer processes between water and the micellar phase calculated by the MD/US method.

## 2 Methods

### 2.1 Synthesis

All chemicals were purchased by Merck and used as received. For the synthesis of methyl [1,1′-biphenyl]-4-acetate, methyl (4-bromophenyl)acetate (0.5 mmol), phenylboronic acid (0.75 mmol), tetrakis(triphenylphosphine)palladium(0) (0.01 mmol), and triethylamine (1.5 mmol) were suspended in 1 mL of 2 wt % solution of Kolliphor EL in water. The mixture was stirred at room temperature overnight and then extracted with AcOEt (5 mL). The organic phase was filtered through a short plug of silica. The plug was further eluted with 5 mL of AcOEt. Solvent evaporation gave the product in quantitative yield as a light yellow solid. Characterizations agreed with those previously reported.^33^

### 2.2 Computational Methods

MD simulations have been performed using the 2016H66 force field.^25^ Parameters of compounds **1**–**4** not available in 2016H66 have been derived from those of the OPLS2005 force field.^34^ In the case of compound **3**, bond distance and angles and dihedral angles have been restrained to the values calculated by optimization at the DFT level (functional M062X;^35^ basis set 6-31G*; for Pd, the LANL2DZ pseudopotential and related basis set). Water molecules have been described by the SPC^36^ model. In the case of compound **2**, the anionic form [PhB(OH)_3_]^−1^ (labeled as **2′** in the following) has been considered, taking into account the basic environment of the reaction.

Calculations have been performed by the Gromacs program (2020.6 revision).^37,38^ MEP atomic charges of compounds **1**–**4** have been calculated by fitting the molecular electrostatic potential (MEP) calculated in a vacuum at the Density Functional Theory level (functional M062X;^35^ basis set 6-31G*; for Pd, the LANL2DZ pseudopotential and related basis set).

Simulations were performed by using periodic boundary conditions. The Verlet cutoff scheme was applied, with a cutoff distance of 1.4 nm for the nonbonded forces, beyond which the Particle-mesh Ewald method (PME) was used for long-range electrostatic forces. The LINCS algorithm^39^ was used for constraining bonds involving hydrogen atoms. Newton’s equations of motion were integrated every 2 fs with the leapfrog algorithm. Two velocity rescaling thermostats,^40^ one for the solute and the other for the solvent, were used for constant temperature simulations; the Parrinello–Rahman barostat^41^ for constant pressure simulations has been used.

#### 2.2.1 The o/w Partition Free Energies

The o/w partition free energies of compounds **1**–**4** have been calculated as the difference between the solvation free energies determined in *n*-octanol and in water by the SMD and MD/FEP methods.

Quantum-mechanical solvation free energies have been calculated with the *Gaussian 16* program^42^ at the DFT level (functional M062X) by the SMD model,^32^ a quantum mechanical method developed to include the solvent effects in the calculation of the energy of a molecular system in solution. Solvent effects are included by describing the solvent as a continuum polarizable medium that affects the electronic distribution of the solute.

MD/FEP solvation free energies have been calculated by using either the MEP atomic charges or, following the MDEC (Molecular Dynamics Electronic Continuum) model,^43,44^ the MEP atomic charges scaled by a 0.7 factor and adding the electronic polarization contribution to the electrostatic term calculated by MD/FEP. The electronic polarization contribution has been calculated by using the PCM model implemented in *Gaussian 16*; in this calculation, the solvent is described by a continuum polarizable model, characterized by its electronic dielectric constant ε_el_, and the solute, with nonscaled atomic charges, is placed into a cavity with the dielectric constant equal to one.

MD/FEP simulations were performed by switching off the electrostatics and van der Waals interactions between the solute and the solvent independently, using a parameter λ. Electrostatics interactions were switched off, while van der Waals interactions were present, in 20 steps (dλ = 0.05), from λ = 0; that is, the solute totally interacts with the solvent, to λ = 1, when only the solute–solvent van der Waals interactions are still present. The latter then were switched off with the atomic charges of the solute set to zero; in most of the cases, decreasing dλ to 0.02 or 0.01 in the region ranging from λ = 0.5 to λ = 1 was necessary to obtain adequate information on the entropy values.^41^ For each λ step, the system was subjected to the following simulation setup: (i) energy minimization; (ii) 100 ps equilibration at constant volume and temperature (298 K), with position restraints applied on the solute; (iii) 100 ps equilibration at constant pressure (1 bar) and temperature (298 K), with position restraints applied on the solute; and (iv) 1 ns production run at constant pressure (1 bar) and temperature (298 K). The free energy of solvation was then calculated with Bennett’s acceptance ratio method (BAR).^45,46^

#### 2.2.2 Micelle Computational Model

Modeling of the micelle started with an exhaustive sampling of the conformational space of a single K-EL molecule. Starting from an initial minimum energy conformation, one K-EL molecule was inserted into a cubic box of 8 nm sides and solvated with about 17 000 water molecules. Energy minimization (steepest descent algorithm) was followed by NVT (200 ps) and NPT equilibration (500 ps), both of which were performed with position restraints applied on the solute. A 50 ns NPT production run was then performed after removal of the position restraints. This procedure was repeated starting from three other different initial conformations. The four trajectories (total simulation time: 200 ns) were then concatenated and analyzed by cluster analysis (Gromos method, RMSD cutoff = 0.5 nm). Centrotypes of the 16 most populated clusters, representative of 70% of the conformational sampling, have then been used for micelle building.

Sixty-eight K-EL molecules, selected among the above-mentioned centrotypes, were inserted into a cubic box of 17 nm sides and solvated with about 302 000 water molecules. This setup corresponds to a K-EL concentration equivalent to the experimental conditions (2 wt %). Energy minimization (steepest descent algorithm), followed by NVT (200 ps) and NPT equilibration (500 ps), with position restraints applied on the solute was performed. After position restraint removal, a 100 ns NPT production run was carried out.

The characterization of the micellar aggregates formed includes: (i) calculation of the radii of gyration (*R*_g_) and the effective micellar sizes (*R*_s_), defined as *R*_s_ = *R*_g_(5/3)^1/2^, based on the assumption that aggregates are quite spherical; and (ii) calculation of the Radial Distribution Function (RDF).

#### 2.2.3 Micelle/Water Partition Free Energy

By using the micelle model obtained in the previous step, we carried out steered MD simulations. Each solute molecule was inserted in the water phase present in the simulation box, and a short NVT–NPT equilibration was run. It then was pushed (pull rate = 0.004 nm ps^–1^) from the water phase to the center of the micelle (initial distance between the centers of mass of the molecule and the micelle was 50 Å), applying a harmonic potential (1000 kJ mol^–1^ nm^–2^) between the centers of mass of the two groups (the solute and the micelle). Configurations for the MD/US simulations were extracted from the steered MD trajectory every 0.2 nm, when the solute was in the bulk water phase, and every 0.1 nm, from the moment when the solute was approaching the micelle. MD/US simulations were run applying an umbrella potential (*k* = 1000 kJ mol^–1^ nm^–2^) for restraining the distance between the center of mass of the molecule and the micelle at the defined value. For each configuration, a 1 ns equilibration step was followed by a 5 ns production run at constant temperature (300 K) and pressure (1 bar). This procedure was repeated for a total of six different starting positions of the solute with respect to the micelle. These starting positions were identified by centering the micelle in a cube and placing the solute at the centers of its faces, that is, in correspondence with the vertices of a regular octahedron (see Figure S4). The Weighted Histogram Analysis Method (WHAM)^47,48^ was used to extract the Potential of Mean Force (PMF) curve.^49^ The partition free energy was thus obtained from the difference between the plateau of the curve corresponding to the water phase and the lowest point in the micelle.

## 3 Results and Discussion

### 3.1 Synthesis

The reaction of methyl (4-bromophenyl)acetate (0.5 mmol) and phenylboronic acid (0.75 mmol) was performed according to our previously reported micellar method, with the only difference being the use of Pd(0)tetrakis instead of a Pd(II) precursor.^4^ As the reaction mechanism requires Pd(0) for the first step (i.e., the oxidative addition), Pd(II) salts could be used as a source of Pd(0), but a reducing agent would be needed in the reaction environment to promote the formation of the active Pd(0). The use of a preformed Pd(0) catalyst ensures that this catalytic species does not need to be formed in situ, and the modeled catalytic species and the experimental one coincide. The reaction is efficient and does not require removal of oxygen from the reaction mixture, notwithstanding the known oxygen sensitivity of palladium tetrakis. The product can be recovered in quantitative yield by extracting the reaction mixture with little AcOEt.

### 3.2 The o/w Partition Free Energies

To verify the reliability of the adopted force field in describing the micelle/water partition of the reactive species, a preliminary investigation has been done on the reliability of this force field in reproducing the partition free energies of the solute species between water and *n*-octanol, the latter chosen as a representative model of the amphiphilic micellar environment. Partition free energies have been calculated from the solvation free energies determined by the SMD and MD/FEP methods, and, due to the lack of experimental values, solvation free energies calculated by the SMD model have been assumed as a reference.

Concerning the calculation of solvation free energies by means of MD simulations, it should be remarked that in the linear response approximation the electronic polarization effects include two additive terms: (i) a nuclear polarization contribution, due to the “slow” nuclear rearrangement of the solvent molecules around the solute, that is described by the MD simulation; and (ii) a fast electronic polarization term, due to the “instantaneous” electronic response of medium to the solute charges. This latter causes an enhancement of the molecular dipole moment, increasing the strength of the electrostatic interactions between the molecules. At the same time, the electrostatic interactions turn out to be reduced by the electronic screening of the medium, as well as by the presence of ionic species (such as those provided by acid or base dissociation) that modify the ionic strength of the solution. This effect is expected to be particularly significant in the case of ionic solutes, as in the case of compound **2′**. In general, in MD simulations, neither the electronic polarization effects, unless a polarizable force field is used, are included, nor the effects of the electronic screening of the medium. Indeed, because these two contributions approximately compensate for each other when high dielectric constant medium (such as water) or neutral solute molecules are considered, even nonpolarizable force fields can provide good solvation free energies. However, this compensation does not hold when ions or ionized groups are considered because the direct Coulomb interaction dominates, and the screening effect is of primary importance. In these cases, the screening effect can be introduced by scaling the atomic charges of the ionic species.^43,44^

Within the MDEC (Molecular Dynamics Electronic Continuum) approach,^43^ medium screening effects and electronic polarization are taken into account in a simple but efficient way: MD simulations are performed using atomic charges scaled by an appropriate factor, and the electronic polarization contribution is calculated separately and added to the MD electrostatic term. In this approach, the electronic screening of the medium is included by scaling the atomic charges by a 1/√(ε_el_) factor: because the electronic dielectric constant, ε_el_, assumes values typically close to 2, the suggested charge scaling factor is 0.7, which is the one we used in our simulations. We calculated the electronic polarization contribution by using the PCM model implemented in *Gaussian 16*. The Poisson–Boltzmann equations are solved for the solvent described by a continuum polarizable model, characterized by the electronic dielectric constant ε_el_, and the solute, with nonscaled atomic charges, placed into a cavity with the dielectric constant equal to one.

The solvation free energies of compounds **1**–**4** have been calculated using (i) MEP atomic charges, without any scaling procedure and neglecting any polarization contribution (MD/FEP approach); and (ii) scaling MEP atomic charges by 0.7 and including the electronic polarization contribution by PCM (MDEC approach). In the case of the PhB(OH)_2_ species, present as the [PhB(OH)_3_]^−1^ anion, MD/FEP solvation free energies have also been calculated using charges scaled by 0.7, without inclusion of the polarization contribution.

**Figure 2:**
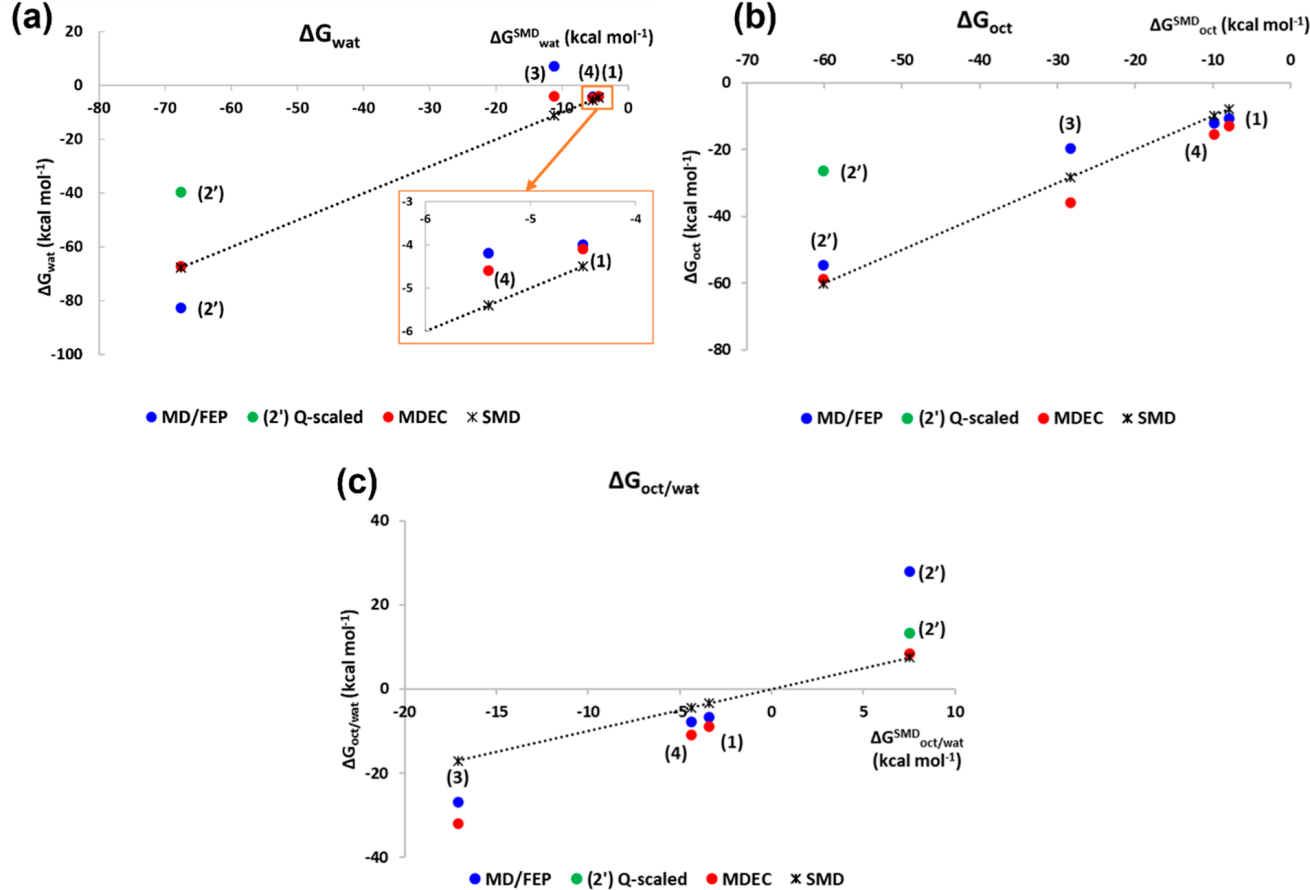
Solvation free energies (kcal mol^–1^) in water (a) and in *n*-octanol (b) and oct/wat partition free energies (c) calculated by MD/FEP (blue) and MDEC (red) versus SMD (*) values. For compound **2′**, scaled charge results are also reported (green point). Dashed line: SMD values.

Solvation free energies together with the resulting *n*-octanol/water partition free energies (calculated as the differences between the solvation free energies) are reported in Table 1 and highlighted in Figure 2. Table S1 reports the electrostatic, van der Waals, and polarization contributions for the MD/FEP and MDEC results.

**Table 1:** Solvation Free Energies in Water and *n*-Octanol (Δ*G*_wat_ and Δ*G*_oct_, kcal mol^–1^) and the Resulting *n*-Octanol/Water Partition Free Energies (Δ*G*_oct/wat_, kcal mol^–1^), Calculated by SMD (M062X Functional), MD/FEP, and MDEC Methods (2016H66 Force Field)

	Δ*G*_wat_			Δ*G*_oct_			Δ*G*_oct/wat_		
	MD/FEP	MDEC	SMD	MD/FEP	MDEC	SMD	MD/FEP	MDEC	SMD
**1**	–4.0	–4.1	–4.5	–10.6	–12.9	–7.9	–6.6	–8.8	–3.4
**2′**	–82.7	–67.2	–67.6	–54.7	–58.7	–60.1	28.0	8.5	7.5
	–39.7^a^			–26.5^a^			13.2^a^		
**3**	7.1	–4.1	–11.2	–19.7	–36.0	–28.3	–26.8	–31.9	–17.1
**4**	–4.2	–4.6	–5.4	–12.0	–15.6	–9.8	–7.8	–11.0	–4.4

a Atomic charges scaled by 0.7.

Comparison with the SMD results shows that in water the MDEC method provides overall more reliable results than MD/FEP, while in *n*-octanol the results are quite comparable. Concerning the *n*-octanol/water partition, both methods provide comparable results, even if they tend to slightly overestimate the lipophilicity of **1**, **3**, and **4** and, mostly, the hydrophilicity of the ionic compound **2′**. Just in the case of compound **2′**, inclusion of the medium screening effect of the electrostatic interactions by scaling of the ion atomic charges appears to be required for the best assessment of the partition free energy.

On the basis of these results, we chose to perform the study of the micelle/water partition by using in the MD/FEP method the standard charges fitted to MEP, apart from the case of the **2′** anion, for which the charges scaled by a 0.7 factor have been used instead.

### 3.3 Micelle Computational Model

**Figure 3:**
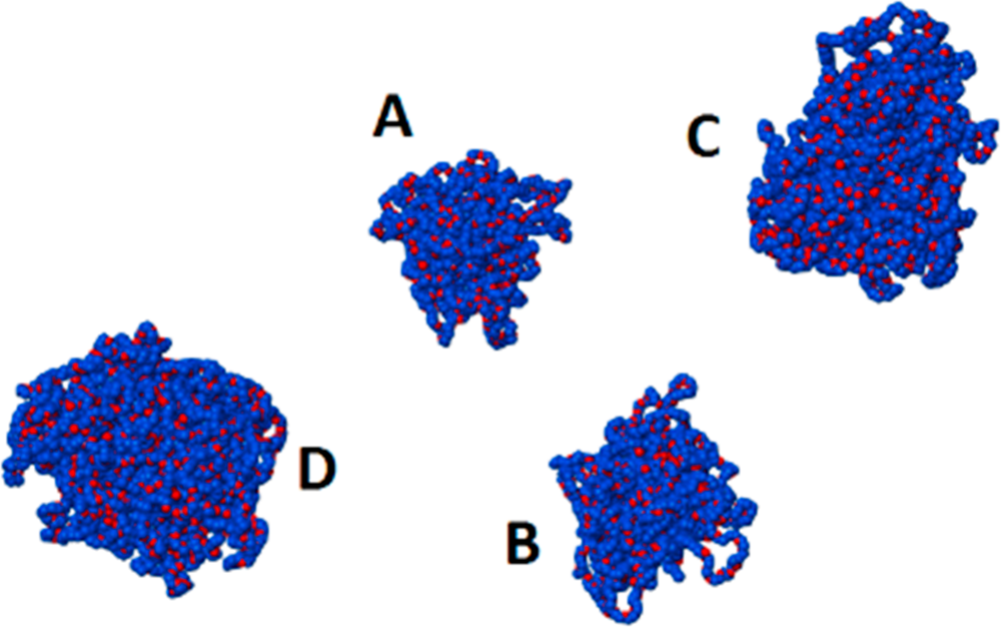
A–D K-EL aggregates after 100 ns of simulation. Carbon atoms are in blue, oxygens are in red, and hydrogens are not shown.

By setting up simulations with 68 K-EL molecules in a 17 nm cubic box size, corresponding to the experimental concentration (2 wt %), we observed the formation of aggregates already after a few nanoseconds; after 70 ns, four aggregates of different sizes are well distinguishable and remain stable for the following 30 ns (Figure 3).

The dimensions of each aggregate, calculated over the last 10 ns of the simulation, are reported in Table 2 in terms of the number of K-EL molecules, the radii of gyration (*R*_g_), and the effective micellar sizes (*R*_s_). The stability radii of gyration in the last 10 ns of simulation of the A–D aggregates (Figure S1) highlight the equilibration of the system, and the *R*_g_ component along the three axes shows that these aggregates present a close-to-spherical shape (Figure S2).

**Table 2:** K-EL Aggregate Dimensional Parameters Calculated over the Last 10 ns of the Simulation

aggregate	number of K-EL molecules	*R*_g_ (nm)	*R*_s_ (nm)
A	10	1.88 ± 0.04	2.43 ± 0.05
B	12	2.11 ± 0.06	2.73 ± 0.08
C	20	2.47 ± 0.02	3.19 ± 0.02
D	24	2.57 ± 0.06	3.31 ± 0.07

Dynamic light scattering (DLS) showed^23^ that a micellar region is detected below 25% w/w, and cryo-TEM measurements on frozen KOL solutions prepared with concentrations of 1.4% and 15% w/w KOL show that the micelle size does not change greatly with concentration. Aggregate D reproduces the K-EL micelle size experimentally determined by cryo-TEM measurements^23^ for a concentration of 1.4 wt %, the latter being close to the one used in the simulations (2 wt %). The calculated diameter of the D aggregate (6.62 ± 0.14 nm) falls within the interval between the minimum (6.0 ± 2.3 nm) and the maximum (8.7 ± 1.7 nm) of the experimental values. Moreover, DLS values of 1.4% and 2% w/w of K-EL in water have been recorded and show no difference in the hydrodynamic volume of the obtained micelles (see Figure S3). Considering that triethylamine is only partially soluble in water at basic pH, it could in principle act as a cosolvent. However, this does not appear to be the case for our reactions. According to the DLS characterization performed on the K-EL 2 wt % dispersion in H_2_O and 1.5 M Et_3_N (see Figure S3), the presence of triethylamine does not significantly influence the hydrodynamic volume of the micelles. On the basis of this result, aggregate D was selected as the model system to represent the micellar environment built up by K-EL in water.

**Figure 4:**
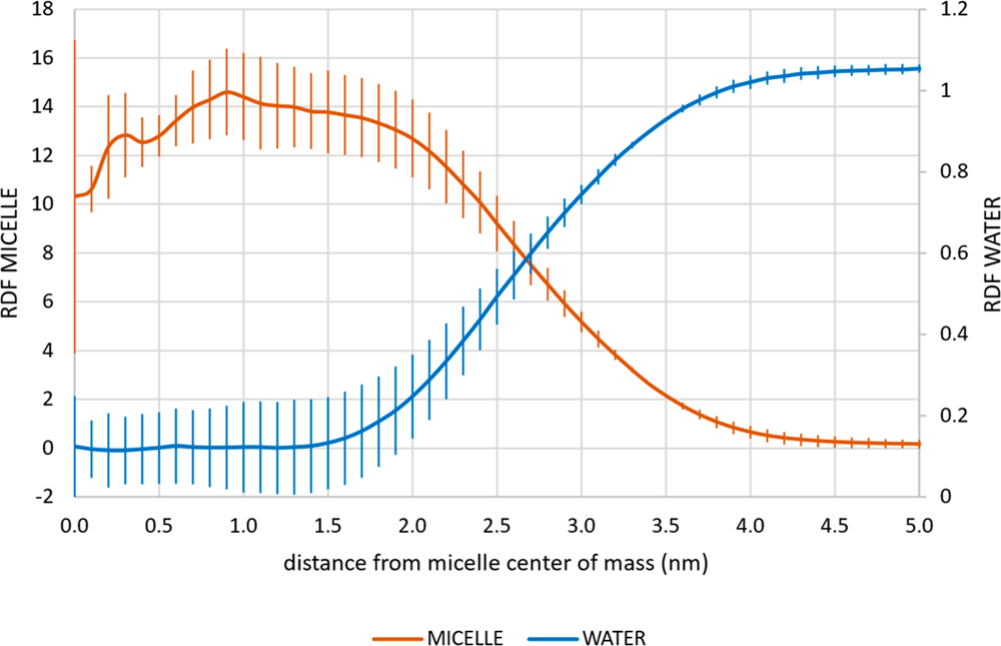
Radial distribution function (average and standard deviations) of the K-EL and water molecules.

On this system, four further MD simulations of 50 ns each were performed to characterize other structural features of this micelle. Figure 4 reports the RDF of the micelle atoms and the RDF of the water molecules, calculated with respect to the center of mass of the micelle. These trends highlight that (i) the center of the micelle is slightly less dense than the region that ranges from 0.5 to 2 nm; (ii) the micelle extends up to 4 nm; and (iii) the micelle is minimally hydrated up to 2 nm, whereas after such a value the hydration level rapidly increases.

**Figure 5:**
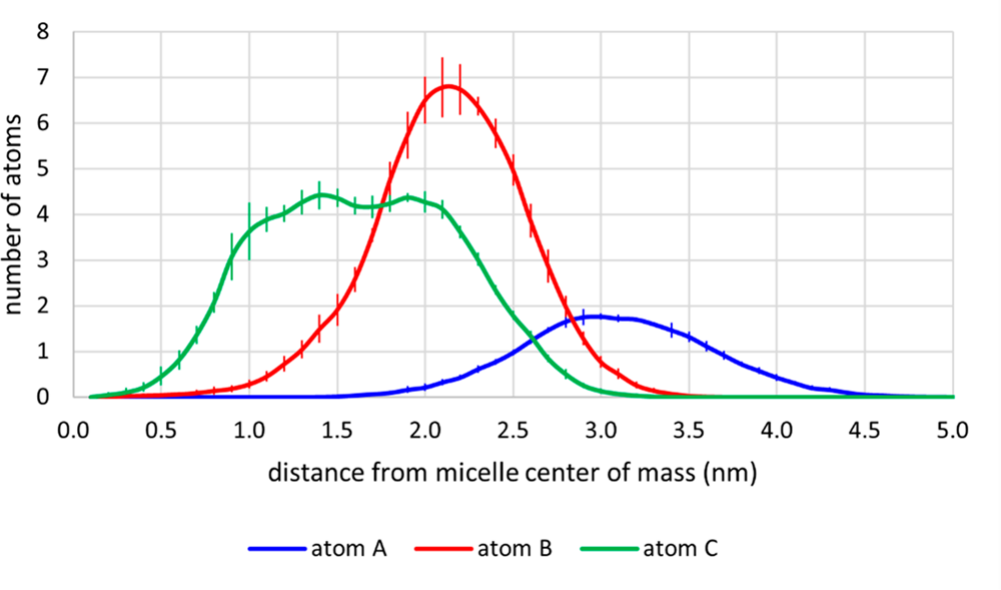
Number (average and standard deviations) of atoms A, B, and C (labeled in Figure 1), selected as representative of the hydrophilic, hydrophilic–hydrophobic interface, and hydrophobic region of K-EL, with respect to the center of mass of micelle D.

Figure 5 shows the number of K-EL atoms A, B, and C, selected as representative (Figure 1) for the hydrophilic, interface hydrophilic–hydrophobic, and hydrophobic regions of the micelle, respectively, with respect to the center of mass of the latter. From this figure, we can infer the organization of the surfactant molecules into the micelle: (i) glycerol moieties (labeled by atom A) are located at the external board of the micelle (from 3.0 to 4.0 nm); (ii) B carbonyl atoms, that is, the interface between the hydrophilic moiety and the hydrophobic tail, span from 1.5 to 3.0 nm; and (iii) C hydroxyls spread a wide region from 1 to 2.5 nm, corresponding to the hydrophobic core of the micelle.

Thus, in agreement with the experimental evidence and with the previous computational micelle model,^23^ aggregate D presents the assembly of the K-EL molecules within the micelle with the fatty acid chains located in the micellar core, thus reproducing the core–shell structure experimentally observed.

### 3.4 Micelle/Water Partition Free Energies

**Figure 6:**
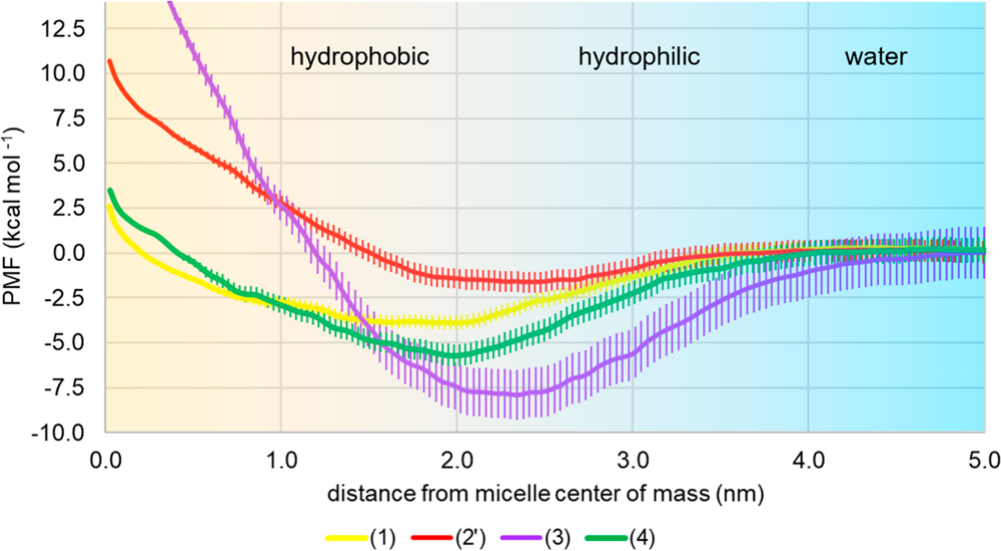
PMF calculated for the four species by MD/US by six independent simulations starting from different positions of the solute with respect to the micelle. Standard deviation bars are reported.

For the calculation of the PMF for the micelle/water partition process of species **1**–**4**, a steered MD simulation followed by MD/US simulations was performed. Because of the intrinsic anisotropy of the micellar system, this procedure was repeated six times, starting from different initial positions of the solute with respect to the micelle, and collecting the overall results. Figure 6 reports for each species the PMF extracted with WHAM from all of the run trajectories.

For reagent **1** and product **4**, the minima in the PMF profile (−3.9 and −5.7 kcal mol^–1^, respectively) are located at around 2 nm, in the region at the boundary between the hydrophobic region and the most hydrophilic part of the micelle. The anionic species [PhB(OH)_3_]^−1^ (**2′**) presents a flat minimum around −1.6 kcal mol^–1^ in the hydrated region ranging from 2 to 3 nm from the micelle center. Finally, the catalyst **3** presents a deeper minimum (−8 kcal mol^–1^) between 2.0 and 2.5 nm. Considering the limited standard deviations of the PMF profiles (<1 kcal mol^–1^ for species **1**, **2′**, and **4**, and <1.5 kcal mol^–1^ for species **3**), we concluded that the number of MD/US procedures carried out was adequate.

As a final remark, the presence of ionic species in solution should provide an increase in ionic strength of the water solution that could result in a more marked preference of the investigated solutes for the micellar phase. Therefore, the values of minima in the PMF profiles should be considered as an upper bound to the partition free energy values.

## 4 Conclusions

Our study concerned the partition of the reactive species involved in the Suzuki–Miyaura reaction between the aqueous and the micellar phases, the latter being constituted by nonionic surfactant K-EL.

A test on the reliability of the adopted force field in describing the *n*-octanol/water partition highlighted the need to take into account the medium screening effects of the electrostatic interactions when ionic species are considered, and the scaling procedure of the atomic charges by the 0.7 factor guarantees the obtainment of sufficiently accurate results.

The micelle model provided by the adopted 2016H66 force field finely reproduces the available experimental features of this system in terms of dimensions, shape, and internal structure.

Finally, the potential of the mean force obtained for the different species by the MD/US approach clearly shows that the micellar portions devoted to becoming the regions where the reaction will take place are those at the interface between the hydrophobic and the hydrophilic regions.

In conclusion, the overall picture that emerges from these results confirms that the molecular species involved in the reaction of interest prefer the micellar environment and concentrate in close zones of the micelle. This supports the experimental evidence that the use of suitable surfactant agents promotes reactivity, allowing the micelles to behave as nanoreactors in which the reactive species are solubilized and enhance their local concentration.

## Supporting Information Available

Table S1, electrostatic, van der Waals, and polarization contributions for the MD/FEP and MDEC results; Figure S1, radii of gyration in the last 10 ns of simulation of the A–D aggregates; Figure S2, components of the radii of gyration along the tree axes for the A–D aggregates; Figure S3, volume distribution of the colloid dimensions in K-EL 1.4 wt % in H_2_O, K-EL 2 wt % in H_2_O, and K-EL 2 wt % in H_2_O in the presence of Et_3_N; and Figure S4, starting positions of compound **1**, reported as an example, considered for the steered MD (PDF)
