# Supplementary material for: Partition of the Reactive Species of the Suzuki–Miyaura Reaction between Aqueous and Micellar Environments
Source: J Phys Chem B. 2022 Nov 4;126(45):9408–16. doi: 10.1021/acs.jpcb.2c04591 (PMC9677424; doi:10.1021/acs.jpcb.2c04591)

Supplementary Information

Partition of Reactive Species of the Suzuki–Miyaura Reaction between Aqueous and Micellar Environment

Anna Ranaudo,[1] Claudio Greco,[1] Giorgio Moro,[2] Anita Zucchi,[3] Sara Mattiello,[3] Luca Beverina,[3] Ugo Cosentino[1]*

*anna.ranaudo@unimib.it

1 Department of Earth and Environmental Sciences – University of Milano-Bicocca, Piazza della Scienza 1, 20126 Milan, Italy

2 Department of Biotechnology and Biosciences – University of Milano-Bicocca, Piazza della Scienza 2, 20126 Milan, Italy

3 Department of Materials Science – University of Milano-Bicocca, Via Roberto Cozzi 55,

20125 Milan, Italy

Table S1. Solvation free energies in water and n-octanol (ΔG_{wat} and ΔG_{oct}, kcal mol^{-1}, standard deviation in parentheses) and resulting n-octanol/water partition free energies ($\Delta G_{oct/wat}$, kcal mol^{-1}), calculated by SMD (M06-2X functional), MD/FEP and MDEC methods (2016H66 force field). Reported electrostatic, van der Waals, and polarization contributions.

		ΔG_{wat}			ΔG_{oct}			$\Delta G_{oct/wat}$		
		MD/FEP	MDEC	SMD	MD/FEP	MDEC	SMD	MD/FEP	MDEC	SMD
(1)	ΔG_{tot}	**-4.0 (0.1)**	**-4.1 (0.1)**	**-4.5**	**-10.6 (0.1)**	**-12.9 (0.1)**	**-7.9**	**-6.6 (0.1)**	**-8.8 (0.1)**	**-3.4**
	Electr.	-4.3 (0.0)	-2.1 (0.0)		-0.9 (0.1)	-0.5 (0.1)				
	VdW	0.3 (0.1)	0.3 (0.1)		-9.7 (0.1)	-9.7 (0.1)				
	ΔG_{pol}		-2.3			-2.7				
(2')	ΔG_{tot}	**-82.7 (0.1)**<br>**-39.7 (0.1)[a]**	**-67.2 (0.1)**	**-67.6**	**-54.7 (0.2)**<br>**-26.5 (0.2)[a]**	**-58.7 (0.2)**	**-60.1**	**28.0 (0.2)**<br>**13.2 (0.2)[a]**	**8.5 (0.2)**	**7.5**
	Electr.	-84.9 (0.1)<br>-41.9 (0.0)[a]	-41.9 (0.0)		-49.0 (0.2)<br>-20.8 (0.1)[a]	-20.8 (0.1)				
	VdW	2.2 (0.1)	2.2 (0.1)		-5.7 (0.1)	-5.7 (0.1)				
	ΔG_{pol}		-27.5			-32.2				

(3)	**ΔG_{tot}**	**7.1 (0.8)**	**-4.1 (0.4)**	**-11.2**	**-19.7 (0.7)**	**-36.0 (0.4)**	**-28.3**	**-26.7 (1.0)**	**-31.9 (0.6)**	**-17.1**
	Electr.	-0.6 (0.7)	-0.3 (0.3)		13.4 (0.6)	9.6 (0.3)				
	VdW	7.7 (0.2)	7.7 (0.2)		-33.1 (0.3)	-33.1 (0.3)				
	ΔG_{pol}		-11.5			-12.5				
(4)	**ΔGtot**	**-4.2 (0.2)**	**-4.6 (0.0)**	**-5.4**	**-12.0 (0.0)**	**-15.6 (0.0)**	**-9.8**	**-7.8 (0.2)**	**-11.0 (0.0)**	**-4.4**
	Electr.	-5.8 (0.0)	-2.9 (0.0)		-0.8 (0.0)	-0.4 (0.0)				
	vdW	1.6 (0.2)	1.6 (0.2)		-11.2 (0.0)	-11.2 (0.0)				
	ΔG^{pol}		-3.3			-4.0				

[(a)]Atomic charges scaled by 0.7.

Figure S1. Radii of gyration in the last 10 ns of simulation of the A-D aggregates.

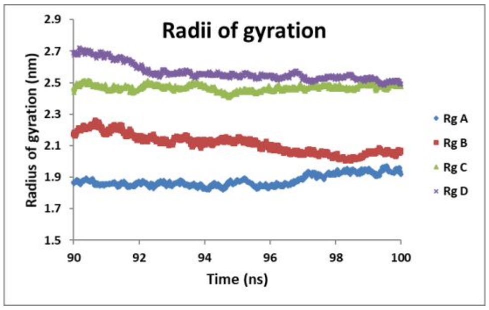

Figure S2. Components of the radii of gyration along the tree axes for the A-D aggregates.

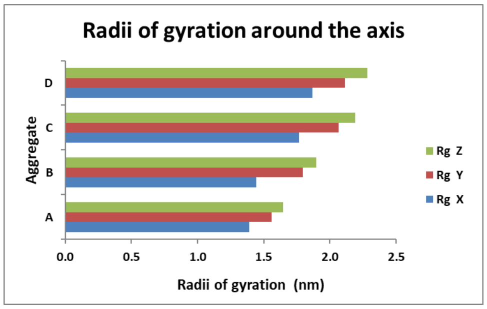

Figure S3. Volume distribution of colloids dimensions in K-EL 1.4 wt% dispersion in H_2O (red line), K-EL 2 wt% dispersion in H_2O (blue line), and K-EL 2 wt% dispersion in H_2O and 1.5 M Et_3N (green line).

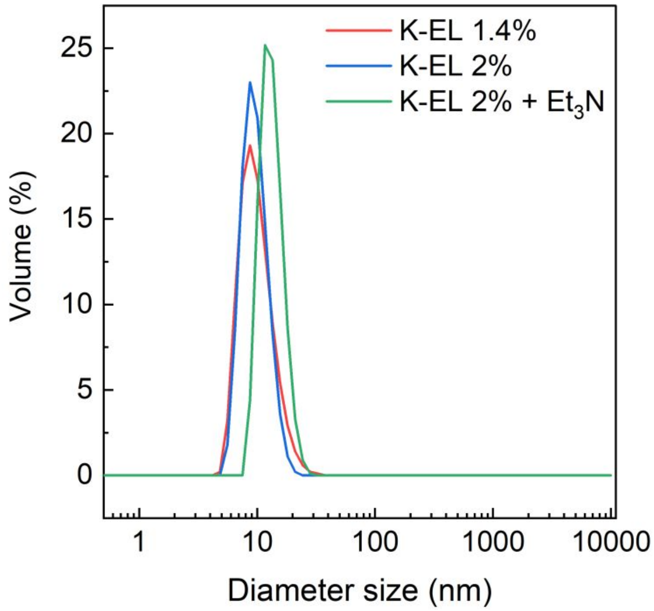

Figure S4. Starting positions of compound (**1**), reported as an example, considered for the steered MD.

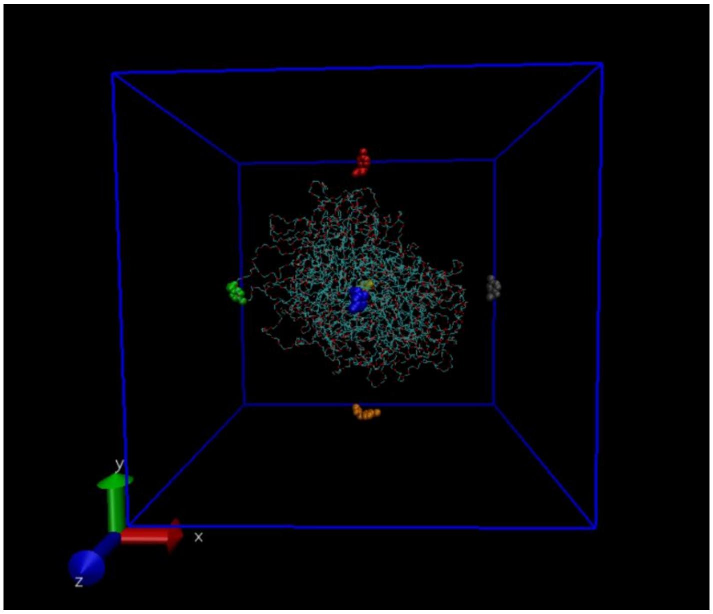

Supplement: Supplementary file 1 — jp2c04591_si_001.pdf [file jp2c04591_si_001.pdf]
